# Active surveillance is a feasible and safe strategy in selected patients with papillary thyroid cancer and suspicious cervical lymph nodes detected after thyroidectomy

**DOI:** 10.20945/2359-4292-2023-0146

**Published:** 2024-05-07

**Authors:** Marlín Solórzano, Nicole Lustig, Lorena Mosso, Martín Espinoza, Roberto Santana, Hernan Gonzalez, Pablo H. Montero, Francisco Cruz, Antonieta Solar, José Miguel Domínguez

**Affiliations:** 1 Pontificia Universidad Católica de Chile Facultad de Medicina Departamento de Endocrinología Santiago Chile Departamento de Endocrinología, Facultad de Medicina, Pontificia Universidad Católica de Chile, Santiago, Chile; 2 Centro de estudios traslacionales de Endocrinología Santiago Chile Centro de estudios traslacionales de Endocrinología (Cetren) UC, Santiago, Chile; 3 Pontificia Universidad Católica de Chile Facultad de Medicina Departamento de Oncología Quirúrgica Santiago Chile Departamento de Oncología Quirúrgica, Facultad de Medicina, Pontificia Universidad Católica de Chile, Santiago, Chile; 4 Pontificia Universidad Católica de Chile Facultad de Medicina Departamento de Radiología Santiago Chile Departamento de Radiología, Facultad de Medicina, Pontificia Universidad Católica de Chile, Santiago, Chile; 5 Pontificia Universidad Católica de Chile Facultad de Medicina Departamento de Patología Santiago Chile Departamento de Patología, Facultad de Medicina, Pontificia Universidad Católica de Chile, Santiago, Chile

**Keywords:** Papillary thyroid carcinoma, locoregional structural disease, active surveillance

## Abstract

**Objective:**

After initial treatment, up to 30% of patients with papillary thyroid cancer (PTC) have incomplete response, mainly cervical lymph node (LN) disease. Previous studies have suggested that active surveillance (AS) is a possible option for these patients. Our aim was to report the results of AS in patients with PTC and cervical LN disease.

**Materials and methods:**

In this retrospective observational study, we included adult patients treated and followed for PTC, who presented with cervical LN disease and were managed with AS. Growth was defined as an increase ≥ 3mm in either diameter.

**Results:**

We included 32 patients: 27 (84.4%) women, age of 39 ± 14 years, all initially treated with total thyroidectomy, and 22 (69%) with therapeutic neck dissection. Cervical LN disease was diagnosed 1 year (0.3-12.6) after initial management, with a diameter of 9.0 mm (6.0-19.0). After a median AS of 4.3 years (0.6-14.1), 4 (12.5%) patients had LN growth: 2 (50%) of whom were surgically removed, 1 (25%) was effectively treated with radiotherapy, and 1 (25%) had a scheduled surgery. Tg increase was the only predictive factor of LN growth evaluated as both the delta Tg (p < 0.0366) and percentage of Tg change (p < 0.0140). None of the included patients died, had local complications due to LN growth or salvage therapy, or developed distant metastases during follow-up.

**Conclusions:**

In selected patients with PTC and suspicious cervical LNs diagnosed after initial treatment, AS is a feasible and safe strategy as it allows effective identification and treatment of the minority of patients who progress.

## INTRODUCTION

After initial treatment of papillary thyroid cancer (PTC), up to 30% of patients have incomplete response, usually as a persistent or recurrent structural disease identified in cervical lymph nodes (LNs) (1). In this scenario, the 2 major concerns are i) the appearance of local complications (nerve injury or invasion into adjacent structures) due to disease progression and ii) the development of distant metastases (1,2).

In this setting, the options of management go from surgery in one extreme to active surveillance (AS) in the other (3,4). In between them, alternatives include radioactive iodine therapy and ethanol or radiofrequency ablation, among others (5,6). Although this clinical scenario is frequent, no prospective randomized studies have compared the different alternatives, and the available information comes from retrospective studies with a relatively small number of patients and a restricted length of follow-up (7-9).

Regarding this issue, the 2015 American Thyroid Association (ATA) guidelines recommend that the best decision is made through a collaborative team approach involving surgeons, endocrinologists, radiologists, the patient, and the family (10). Several observational studies have suggested that low-volume locoregional structural disease has an indolent course, with rates of progression between 9% and 35%, and can be managed through AS (8,9,11,12).

Our goals were i) to describe the frequency of locoregional growth and distant metastasis appearance in a group of patients with PTC and suspicious cervical LNs managed with AS and ii) to identify predictive factors for these outcomes.

## MATERIALS AND METHODS

We conducted a retrospective observational study in the thyroid cancer clinic at *Pontificia Universidad Católica de Chile*, Santiago, Chile. All patients with thyroid cancer were identified from an institutional database of 1,402 patients followed in our center. We included patients aged 18 years or older with PTC and locoregional structural disease who had at least 1 neck ultrasound (US) study during their follow-up between December 2012 and November 2022, were followed for a minimum of 6 months, and were judged by our team to be at low risk of rapid disease progression according to the following inclusion and exclusion criteria. Inclusion criteria included the following: i) cervical LNs ≥ 5 mm in the smallest diameter with a fine needle aspiration (FNA) compatible with PTC (cytologic confirmation or Tg in the aspirate fluid > 10 ng/dL); and/or ii) the presence of at least 2 suspicious US features (hypoechogenicity, rounded shape, microcalcifications, cystic component, absence of hilum, hypervascularity, and hyperechogenic tissue, similar to that seen in the thyroid). FNA was indicated when its result was expected to change the clinical conduit. However, some patients preferred FNA to be performed to confirm or discard malignancy regardless of the choice of treatment and some were referred to us after the FNA was done. We excluded patients with LNs ≥ 20 mm in the largest diameter, 18F FDG PET (+) disease (SUV max ≥ 5), known history of rapid disease progression the 6 months before initial evaluation, and concern for potential complications from local invasion or unsolved distant metastases.

Patients were classified according to the ATA 2015 recurrence risk category (low, intermediate, and high) as well as the eighth edition of the AJCC/UICC staging system (I, II, III, and IV) based on the preoperative neck US, intraoperative findings, and final surgical pathology report (3,13).

All patients were treated with a levothyroxine dose to maintain a serum TSH < 0.1 or between 0.1 and 0.5 uUI/L, depending on the presence of risk factors for thyrotoxicosis-related complications (age, atrial fibrillation, osteopenia, etc.) (3). The patients were followed with clinical examination, thyroglobulin (Tg), antithyroglobulin antibodies (TgAb), and neck US every 6 months during the first year and thereafter at 6- to 12-month intervals according to their response to treatment. Head and neck radiologists performed neck USs. Additional images were performed at the attending physician´s discretion.

Tg was measured using a chemiluminescent immunoassay with a functional sensitivity of 0.1 ng/mL (Elecsys II, Roche Diagnostics, Rotkreutz, Switzerland). TgAb was measured with a chemiluminescent immunoassay (Architect i1000, Abbott Laboratories, Abbott Park, IL), with a reference value of up to 4.11 IU/mL and an analytical sensitivity of 1.0 IU/mL.

Study entry was defined as the time of the first US showing suspicious LN. The study period was defined as the time from the study entry to i) the last available US obtained as part of AS, ii) the time of LN growth, or iii) the time the patient decided to receive further treatment even when LN had no progression.

The primary endpoint was LN growth during follow-up. Growth was defined as an increase of at least 3 mm in any dimension when compared with the size at the time of study entry.

As predictive factors of structural progression, we evaluated the role of clinicopathologic presentation at diagnosis of PTC and Tg and TgAb behavior during follow-up.

Categorical variables are expressed as number and percent; continuous variables are presented as mean and standard deviation, or median and range, as appropriate. Categorical comparisons were performed using Fisher’s exact test, and continuous variables were compared using Student’s t-test for parametric variables and the Mann-Whitney U test for nonparametric variables. Normal distribution of continuous variables was assessed using the Shapiro-Wilk normality test. We explored the relation between various clinicopathological variables (sex, age, histology, risk of recurrence and largest LN diameter) and LN growth: We also studied the relation between initial, final, delta (calculated as final-initial) and percentage of variation ((calculated as (final-initial)/initial)) of Tg and TgAb concentrations and LN growth. A p-value of < 0.05 was considered significant. Statistical analysis was performed using SPSS (v.21.0.0: SPSS, Inc., Chicago, IL).

The study was approved by the institution’s Ethics Committee (CEC-MedUC 13-400) and was performed in accordance with the Declaration of Helsinki (2013).

## RESULTS

We included 32 patients comprising 27 (84.4%) females, with a mean age of 39 ± 14 years and a median time of follow up since PTC diagnosis of 6.1 years (1.9-19.4). All the patients were initially treated with total thyroidectomy, and 22 (69%) were additionally treated with therapeutic neck dissection. RAI ablation was performed in 30 (94%) of the patients at initial management and received a mean dose of 115 ± 42 mCi. Most patients (62.5%) had classical subtype PTC, and 37.5% had other subtypes, including 2 (6.25%) patients with the tall cell subtype and 2 (6.25%) with the hobnail subtype (Table 1). Most patients (81.3%) were classified as low or intermediate risk, but 6 (18.7%) had high risk of recurrence: 5 patients had N1 status > 30 mm, and 1 had an isolated sub-centimetric lung metastasis that grew from 3 mm to 9 mm and was effectively treated with surgery before enrollment in our study.

**Table 1:** Baseline characteristics of patients included in the study (n = 32)

Variable		Total cohort N = 32	LN disease confirmed N = 18	LN disease presumed N = 14
Age at PTC diagnosis (years)		36.1 (18-70)	37.0 (22-70)	36.0 (18-54)
Sex				
	Women	27 (84.4%)	17 (94.4%)	10 (71.4%)
Surgery				
	Total thyroidectomy	10 (31%)	5 (27.8%)	5 (35.7%)
	Total thyroidectomy + neck dissection	22 (69%)	13 (72.2%)	9 (64.3%)
Radioiodine treatment (I-131)				
	Yes	30 (94%)	18 (100%)	12 (86%)
Initial diameter of LNs in AS (mm)		10 (6-19)	9 (6-16)	9 (6-19)
Subtype				
	PTC Usual	20 (62.5%)	11 (61.1%)	9 (64.3%)
	PTC Usual + follicular subtype	8 (25%)	4 (22.2%)	4 (28.6%)
	PTC Hobnail subtype	2 (6.25%)	1 (5.6%)	1 (7.1%)
	PTC Tall cells subtype	2 (6.25%)	2 (11.1%)	0 (0%)
Risk of recurrence ATA 2015				
	Low	1 (3.2%)	0 (0%)	1 (7.1%)
	Intermediate	25 (78.1%)	15 (83.3%)	10 (71.4%)
	High	6 (18.7%)	3 (17.7%)	3 (21.4%)
AJCC 8th edition staging system				
	I	28 (87.5%)	14 (77.8%)	14 (100%)
	II	2 (6.3%)	2 (11.1%)	0 (0%)
	III	1 (3.1%)	1 (5.6%)	0 (0%)
	IVB	1 (3.1%)	1 (5.6%)	0 (0%)
Frequency of locoregional disease progression		4 (12.5%)	3 (16.7%)	1 (7.1%)

PTC: papillary thyroid carcinoma; AJCC: American Joint Committee on Cancer; ATA: American Thyroid.

LN disease was diagnosed with a median of 1 year (0.3-12.6) after initial management. All the included patients had 1 suspicious LN, with a median largest diameter of 9.0 mm (6-19). Cytologic confirmation of a disease with FNA was performed in 18 (56%) patients. There were no clinicopathologic or US differences between patients who had cytologic confirmation and those who did not (Table 1).

AS extended for a median of 4.3 years (0.6-14.1), 4 (12.5%) patients had LN growth, and none of them had growth-related complications (Table 2). Of them, 3 had LN confirmed disease and 1 had presumed LN disease. The frequencies of LN growth were not different between patients with LN confirmed and LN presumed disease (16.7% *vs.* 7.1%, p = 0.406) (Table 1). From the 4 patients who had LN growth, 2 were surgically removed and received an additional dose of RAI, 1 was treated with external beam radiotherapy because she was deemed to have a high risk of surgical complications, and 1 patient was awaiting surgery due to insurance issues. The 3 treated patients accomplished structural control of the disease, without evidence of structural disease on neck US after a median of 2.6 years (0.9-2.9) of follow-up after salvage therapy and had no treatment-related complications. In the patient awaiting surgery, the LN had remained stable 6 months after the diagnosis of LN growth.

**Table 2:** Follow-up of patients included in the study (n = 32)

Variable	Total n = 32
Follow-up time (years)	6.1 (1.9-19.4)
Time until structural disease diagnosis (years)	1.0 (0.3-12.6)
AS length (years)	4.3 (0.6-14.1)
Locoregional disease progression	
Yes	4 (12.5%)

AS: active surveillance.

**Figure 1:**
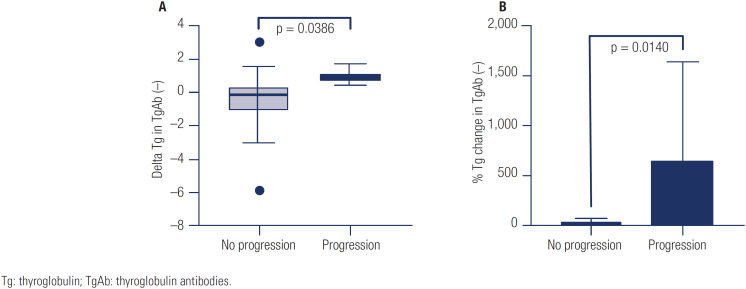
Univariate analysis for factors predictive of lymph node growth (progression).

Among the included variables analyzed (sex, age, histology, risk of recurrence, and initial and follow-up Tg and TgAb concentrations), the only predictive factor for LN growth was the increase of Tg evaluated as both the delta Tg (p < 0.0366) and percentage of Tg change (p < 0.0140) in patients with negative TgAb (Figure 1).

Of the 28 (87.5%) in whom the LN disease remained stable, 6 (19%) patients received some treatment due to their own preference: all of them were subjected to surgery, and 1 additionally received RAI. There were no deaths in the whole cohort, there were no growth- or treatment-related complications, and none of the patients developed distant metastasis during follow-up.

## DISCUSSION

In this study, we found that AS is a feasible and safe strategy in selected patients with PTC and suspicious locoregional LNs. In our series, only 12.5% of patients had structural progression, defined as an increase of at least 3 mm in any dimension during follow-up. There were no local complications related to LN growth or appearances of distant metastases, and no patients in this cohort died. In addition, in cases of disease progression, salvage therapy achieved effective control of locoregional structural disease, without treatment-related complications.

Our results suggest that patients with PTC and suspicious locoregional LNs up to 20 mm of the largest diameter, 18F FDG PET (-), and without history of rapid disease progression can be followed with AS. Our findings are consistent with previous studies that have reported LN growth rates between 14% and 24% (8,9,11,14). All of these series included patients with a profile similar to ours, although there are some interesting differences. Considering that one of the concerns in this setting is the rapid growth and invasion into adjacent structures, some series excluded patients with aggressive histologic subtypes (7,8). In our series, 4 (12.6%) patients had tall cell or hobnail subtype PTC and were not excluded because 3 (75%) initially had intermediate risk of recurrence solely because of the subtype, and the other presented with a single sub-centimeter lung metastasis that was effectively treated with surgery. In this regard, recent evidence has shown that lobectomy is an effective strategy of treatment in T1/T2 and N0 tall cell subtype PTC, thus suggesting that the sole presence of an aggressive histology should not always induce a more active treatment strategy (15).

Because another major concern is the potential relation between locoregional disease and the development of distant metastases (1,9), some studies excluded patients who initially had distant disease (8,11), whereas others included them (9). In this study, we included patients who had been effectively treated for distant metastases, and even though we only had 1 patient fulfilling these criteria, no patients of this cohort developed distant metastases during follow-up. Furthermore, like other authors, we found no association between the presence of suspicious locoregional LNs, their growth, or treatment with surgery, RAI or radiotherapy, and development of distant metastases (8,9,11).

As it is of clinical relevance, we looked for predictors of structural progression and found that none of the studied clinicopathologic features at initial management of CPT were associated with LN growth. However, we found that the increase in Tg during follow-up was associated with LN growth. This is consistent with the findings of Robenshtok and cols. and Jerkovich and cols., who found that a Tg increase ≥ 0.5 ng/mL during follow-up was predictive of LN growth (8,9). Although in other series the frequency of patients presenting with increased Tg and LN growth was only near 20%, thus reflecting a restricted sensitivity, we believe that the Tg trend could be a useful tool when following these patients and, for example, may guide the frequency and amplitude of imaging studies (9).

When following locoregional disease, although one of the concerns is missing the opportunity to effectively treat these patients, we found that, in cases of LN growth, salvage therapy achieves a good disease control. In our series, 3 patients were effectively treated: 2 with surgery and 1 with radiotherapy. In a fourth patient with disease progression who had to delay surgery, the LN remained stable for 6 months. This last finding is consistent with the results from Robenshtok and cols. and Tomoda and cols., who found a slow rate of progression when LN grew (medians of 1.4 and 1.5 mm/year) (9,11).

The main goals of cancer treatment are to improve survival and recurrence (16). Fortunately, thyroid cancer-associated mortality is low, so the preservation of quality of life (QoL) emerges as a very important issue (17). In patients with PTC and locoregional structural disease, there are several management options, from surgery to AS (3,18). Between both there are noninvasive techniques such as radiofrequency ablation and ethanol ablation (5). All of them have related costs and complications. No controlled studies have compared the different therapeutic approaches, and the decision must be made considering the initial presentation of PTC, size, rate of growth, location of the LN, and patient preference (1,18). It has been suggested that, to balance disease control and preserve QoL, surgery is the best approach for patients with a larger and more aggressive disease, whereas AS is the appropriate choice for patients with small volume and least aggressive PTC (3). Noninvasive techniques could be a good option in surgical candidates who have high risk of complications due to local factors or comorbidities, and in maximalist patients with a nonaggressive disease who require an active treatment (19).

Although ATA guidelines recommend not performing FNA in neck LNs to confirm malignancy if the result will not lead to additional evaluation or treatment, it is still controversial because some series only included patients who had cytologic disease confirmation, whereas others included both patients with FNA or ultrasonographic suspicious LNs (7-9,11). As it is our daily practice, we included in our series patients with confirmed and presumed cervical LN disease. Both groups were similar regarding initial risk of recurrence, AJCC/UICC staging system, size of LN, and frequency of LN growth (7.1% *vs.* 16.7%, p = 0.613). Regarding the lack of statistical difference in LN growth between both groups, it may be due to the number of patients included in our series, since in the study from Robenshtok and cols., the LN growth ≥ 5 mm frequencies were 18% *vs.* 9% (9). From a clinical standpoint, it is interesting that when analyzing the data from obtained from Robenshtok and cols., which includes patients with presumed and confirmed cervical LN, and Tomoda and cols. and Jerkovich and cols., which exclusively include confirmed cervical LN, all studies found similar frequencies of LN growth ≥ 3 mm (20-24%) and ≥ 5 mm (8.0-9.0%) (8,9,11). These findings support the ATA recommendation regarding the lack of cytologic confirmation requirement to enroll patients in an AS program (3,20).

Our study has several strengths. First, the inclusion of patients with and without cytological confirmation ensures that ours is a real-world study because it represents what most clinicians do and how guidelines suggest proceeding in this setting (3). Another strength is the cautious inclusion of aggressive histology PTC (tall cell and hobnail subtype) because it demonstrates that it may not be appropriate to make a decision based solely on the presence of a single risk factor (15). Finally, although the inclusion criteria restricted the amount of PTC deemed appropriate to AS, it allowed the selection of a cohort of patients in whom AS is feasible and safe.

The main limitations of this study are its retrospective nature and the small size of the cohort, as with most studies in the field of PTC. However, all patients who met the inclusion criteria were followed prospectively and treated following the same protocol and according to current ATA and local guidelines (3,21). Another limitation may be the relatively short length of follow-up (median of 4.3 years), although it is longer than previous published series in the field (7-9,11).

In conclusion, in selected patients with PTC and incomplete response due to suspicious cervical LN disease, AS is a feasible and safe strategy as it allows professionals to identify and effectively treat the minority of patients who progress, and it is not associated with locoregional complications, development of metastatic disease, or PTC-related death. The trend of Tg concentration during follow-up may be used as a tool to predict LN growth.
